# A FERM-adjacent (FA) region defines a subset of the 4.1 superfamily and is a potential regulator of FERM domain function

**DOI:** 10.1186/1471-2164-7-85

**Published:** 2006-04-20

**Authors:** Anthony J Baines

**Affiliations:** 1 Department of Biosciences, University of Kent, Canterbury, CT2 7NJ, UK

## Abstract

**Background:**

Proteins containing FERM domains comprise a diverse group of eukaryotic proteins that bind membrane proteins and lipids. In doing so, they organise membrane microstructure, and coordinate the disposition of signalling and cell adhesion complexes. In protein 4.1R, phosphorylation adjacent to the FERM domain regulates its activity, and membrane mechanical properties.

**Results:**

A novel sequence domain has been detected in a subset of proteins that contain FERM domains. This subset includes the true 4.1 proteins, some tyrosine phosphatases, rho-GEF proteins and type II transmembrane proteins, as well as some uncharacterised FERM proteins. This FERM-adjacent region is always closely proximate to the C-terminal of the FERM domain. This sequence is likely to be folded with elements of α and β structure. The FERM-adjacent region of 4.1R contains serine residues phosphorylated by PKC and PKA; these appear conserved in about half of all other FERM-adjacent regions. Phylogenetic analyses indicate that all proteins containing a FERM-adjacent region arose from a single ancestor after FERM domains had started to proliferate in genomes of animals, plants and mycetozoa.

**Conclusion:**

The FERM-adjacent region defines a subset of the FERM proteins in animals. The conservation of motifs in this region that are potential substrates for kinases together with the known regulatory phosphorylation of 4.1R in this region raises the possibility that the FERM-adjacent region is a regulatory adaptation in this subset of the FERM proteins.

## Introduction

FERM domains define the band 4.1 superfamily [1]. The domain takes its name from the 4.1 (four point one) and ERM (ezrin, radixin, moesin) proteins where it was first discovered [1], but many metazoan cytoplasmic proteins that associate with membranes contain FERM domains: such proteins include merlin, talin, KRIT1, the uncoventional myosins VIIA, X and XV, certain non-receptor protein tyrosine kinases (e.g. the FAK and JAK kinases) and phosphatases (e.g. PTP-E1 and PTP-H1). A few examples of FERM domains are also found in mycetozoa and plants. This family is of great interest from several points of view. Several members of the family are tumor suppressors (4.1R, 4.1B, merlin) [2]. More generally, this family carries functions that reflect many of the distinctive features of eukaryotic – and most especially animal – life, including tissue-specific signalling through organisation of membrane domains, mechano-protection of membranes from the stresses of animal movement and participation in the formation of complex tissues through cell-cell and cell-matrix junctions [3].

FERM domains have three-lobed 'cloverleaf' structures; each lobe represents a compactly folded structure. Lobe A (the most N-terminal) has a fold resembling ubiquitin; lobe B (the central lobe) resembles acyl-CoA binding proteins; and lobe C (the most C-terminal) has a fold related to pleckstrin homology domain/phosphotyrosine-binding domain (PTB) [4-10]. The close packing of these domains suggests they do not function independently, but rather form a co-ordinated structure.

FERM domains bind a variety of protein and lipid ligands. For example, in 4.1R, lobe A binds the anion exchanger AE1 (band 3), lobe B binds the PDZ and guanylate kinase protein p55 and lobe C binds glycophorin C [6]. The motif YKRS in Lobe C is required for phosphatidylserine (PS) binding to 4.1R; this motif is required for correct intracellular targeting of 4.1R [11]. In the ERM proteins, the head group of PIP_2 _binds a basic cleft between lobes A and C; this binding displaces the ERM tail from the FERM domain [4] thus unmasking the binding site for cell adhesion molecules (such as ICAM1-3 and L1) on lobe C [12].

The first observation of what is now known as the FERM domain came when Leto et al. [13] subjected 4.1R to limited chymotryptic proteolysis. The FERM domain was released as a 30 kDa fragment. Another protease-resistant fragment released in this experiment was 16 kDa. This region lies between the FERM domain and another important functional domain, the spectrin-actin binding domain. The 16 kDa fragment contains residues phosphorylated by PKA and PKC [14,15]. Importantly, PKC phosphorylation of a serine residue in this region modulates membrane mechanical properties by controlling the activities of both the FERM and the spectrin-actin-binding domain [16].

Mammals have four "true" 4.1 proteins: 4.1R, 4.1N, 4.1G and 4.1B. Sequence alignment revels considerable identity between the N-terminal half of their 16 kDa regions, although the C-terminal halves are much less conserved [17]. Here, I investigate the nature of the conserved part of the 16 kDa region. I report that sequences strongly similar to the conserved part of the 16 kDa region are present in a subset of the 4.1 superfamily. This region seems to form a discrete FERM-adjacent region, with the potential to regulate the activities of its neighbouring FERM domain.

## Results and discussion

### Identification of the FERM-adjacent region

The FERM domain in human protein 4.1R [Swiss-Prot:41_HUMAN] is now defined as residues 285–488 by X-ray crystallography [PDB:1GG3] [6]. The 16 kDa fragment lies directly adjacent to the FERM domain: residues 494–614.

Sequence alignment of the four mammalian "true" 4.1 proteins, 4.1R, 4.1B, 4.1G and 4.1N, reveals that a high level of sequence identity extends beyond the end of the FERM domain, and into the 16 kDa region [17]. To explore this further, I compared the 16 kDa region of human 4.1R with the sequences of other mammalian 4.1R, 4.1G, 4.1N and 4.1B proteins available in the Uniprot Knowledgebase (Swis-Prot/Trembl) [18]. This revealed a region of strong conservation over approximately 60 amino acid residues at the N-terminus of the 16 kDa region. 60 amino acids is a large enough region to fold, and is similar in size to known folding structures such as the SH3 domain.

**Figure 1:**
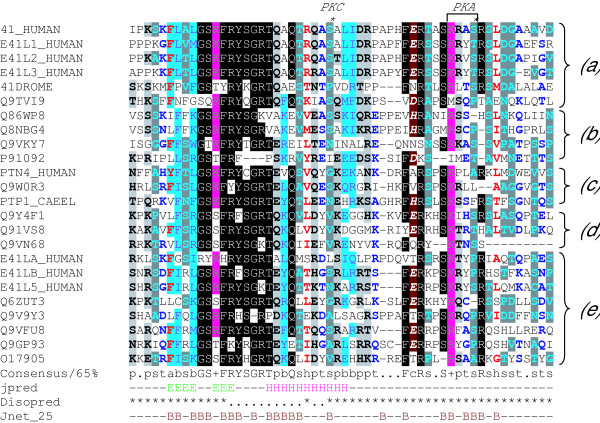
**The FERM-adjacent (FA) region**. An HMM for the FA region was used to search the Uniprot non-redundant database. The alignment shown here represents sequences detected with the HMM in human, *D. melanogaster *and *C. elegans*. The alignment was generated by aligning the sequences to the HMM using HMMALIGN. The sequences shown are all adjacent to the C-terminal end of FERM domains, but not all FERM domains are adjacent to this sequence. Note the presence of a protein kinase A consensus sequence [KR][KR][X][ST] (boxed) in human protein 4.1R (41_HUMAN): the serine (asterisked) in this motif is a physiological PKA substrate. A PKC substrate site is also marked. In 4.1G (E41L2_HUMAN) another serine is phosphorylated in vivo (white asterisk). Secondary structure predictions generated are shown below the alignment. Jpred: E beta-strand; H alpha-helix. Disopred: * disorder. Jnet: B inaccessible to water. The proteins are grouped according to the classes shown in Fig. 2.

To explore the prevalence of this region, the aligned sequences were used to make a hidden Markov model (HMM) that can be used as a profile of the alignment for database searching. An HMM search of Uniprot SPTR revealed further proteins with analogous sequences. These sequences were retrieved, and incorporated into the alignment with the true 4.1 proteins. This expanded alignment was used to generate a new HMM which was used to re-search the database. A third iteration of align sequences/build HMM/search database was performed. The HMM from this reveals no more significant database hits. The full list of sequences detected in given in Table 1 (Additional file 1). Some representative sequences detected by the expanded HMM are shown in Fig. 1.

Strikingly, all the sequences recognised by the HMM are immediately adjacent to a FERM domain. However, they are clearly distinct from FERM domains in that the HMM does not recognise the sequence of any known FERM domain. The HMM detects no proteins whose structures are represented in the Protein Databank. For simplicity, I shall refer to sequences recognised by the HMM as FERM-adjacent (FA) regions.

**Figure 2:**
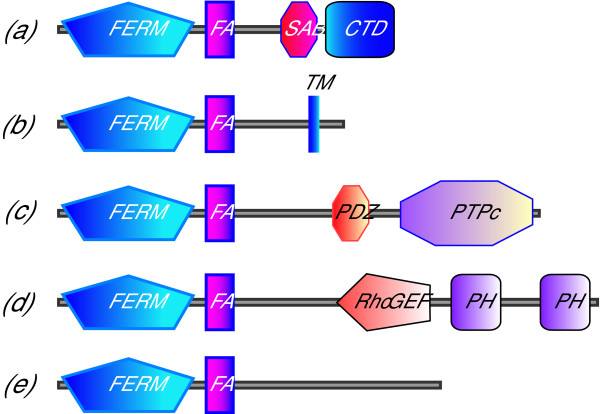
**Examples of classes of proteins containing the FA region**. The figure shows the modular organisation of FA region proteins in schematic outline. The proteins all contain FERM domains in their N-terminal region (although splice variation at certain N-termini can add extra sequence upstream of the FERM domain). On the C-terminal side of the FERM and FA regions are diverse sequences. Examples shown are as follows. (a) The true 4.1 proteins have a C-terminal domain (CTD) that binds receptors; and in mammals a spectrin-actin binding domain (SAB) promotes the binding of spectrin to actin. There are four such in vertebrate genomes, and one such in invertebrate genomes. (b) A group of poorly characterised proteins (two in vertebrate genomes, one in invertebrates) have FERM and FA regions, plus transmembrane segments in their C-terminal regions. They have no N-terminal signal sequences, and appear to be Type II membrane proteins. (c) The non-receptor tyrosine phosphatase protein PTP-MEG1 in mammals, PTP-MEG (split central complex) in fly and protein-tyrosine phosphatase 1 in worm are non-receptor tyrosine phosphatases localised at membranes; they have roles in control of cellular proliferation and control of interaction with extracellular matrix. Notably, the FA region was not found in the other FERM domain phosphatases PTPH1, PTP-E1 and PTP36. (d) CDEP is a GEF for Rho family GTPases. The FA region is also found in the related FARP2 gene product. (e) A variety of other proteins lack further domains recognised by SMART or Pfam. These include Band 4.1-like protein 5 and NBL-4.

The HMM hits include only a minority of members of the 4.1 superfamily. The hits include: (a) the "true" 4.1 proteins; (b) a poorly characterised group of proteins with signal-anchor transmembrane sequences close to their C-termini; (c) a subset of the non-receptor tyrosine phosphatases (e.g. PTP1 of *C. elegans*, and human PTN4); (d) CDEP, a GEF for Rho family GTPases; (e) a number of proteins with only FERM and FA regions, including NBL4 [Swiss-Prot:E41LA_HUMAN] and E41L5 [Swiss-Prot:E41L5_HUMAN]. The domain structures of these proteins are summarised in Fig. 2.

All the sequences detected are in animal proteins with one exception. [Trembl:Q8GUI3] is an *Arabidopsis thaliana *hypothetical protein which contains a FERM domain, and a weak hit (E = 0.00042) is detected adjacent to this. No other plant or mycetozoan protein is detected.

Importantly, many well-characterised members of the band 4.1 superfamily are not detected by the HMM: in particular the ERM proteins, merlin, talin, unconventional myosins and non-receptor protein tyrosine kinases were not found.

A more extended alignment of several FERM proteins that contain the FA region in comparison with representative proteins that do not contain the FA region illustrates the limits of the FA region. Additional file 2 shows the sequences of several human proteins aligned to the sequence of 4.1R Lobe B of the FERM domain through the whole 16 kDa sequence. Note that the FA region is detected as a discrete sequence region corresponding to the N-terminal half of the 16 kDa fragment in class A-E proteins, and that no sequence similarity is detected in other proteins.

### Is the FA region folded?

FA regions seem likely to be compactly folded since the 4.1R 16 kDa fragment is resistant to chymotrypsin [13], despite containing several possible substrate residues (see sequence 41_HUMAN in Fig. 1).

To predict secondary structures, the alignment of all FAs was submitted to JPRED; 4.1R sequence was also submitted to PSI-Pred and Disopred. An α-helix and β-strand and a certain amount of disordered structure are predicted, and the most conserved part of the sequence in the FA alignment is predicted to be buried (annotated on Fig. 1). But the structure cannot be predicted with certainty, because the HMM recognises no sequences of proteins of known structure in the Protein Data Bank. Furthermore, the fold-recognition programmes PSI-Pred and 3D-PSSM do not detect a known fold.

### A single evolutionary event links FERM and FA proteins

The presence of a single FA region in a subset of all FERM domain proteins raises the question of their evolutionary origin. Do these proteins derive from a single ancestral FERM protein that acquired an FA region at some point after FERM domains started to multiply in animal, plant and mycetozoan genomes? If the answer to this question is "yes", then the FA-containing subset of the superfamily should have FERM domains more closely related to each other than to non-FA-containing proteins.

**Figure 3:**
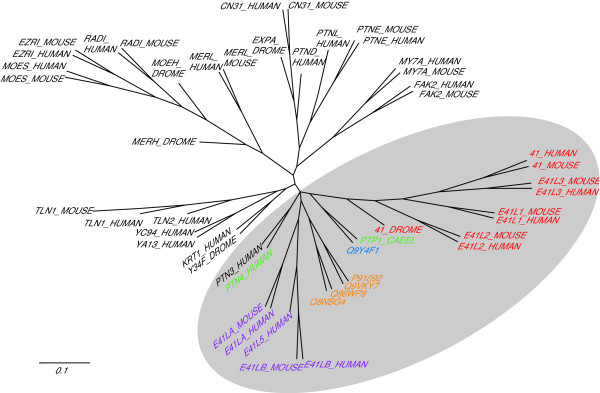
**Phylogenetic tree of lobe B of FERM domains**. This tree was produced by Maximum Likelihood (Phylip proml) analysis of a non-redundant selection of FERM domain lobe B sequences. The grey shading lies over proteins that contain both FERM and FA regions: proteins outside the shading contain FERM domains, but not the FA. FERM/FA region proteins are colour coded to indicate their class of sequence as given in Fig 2: (a) red (b) orange (c) green (d) blue (e) purple.

To address this, a structurally well-conserved part of the FERM domain, lobe B, was subjected to phylogenetic analysis. To define sequences homologous to lobe B, the sequences of lobe B that are defined structurally in the PDB (from protein 4.1R, merlin, ezrin, radxin, moesin, talin) were used to make another HMM specific for lobe B. Searching Uniprot with this HMM defined lobe B in the majority of known members of the superfamily. The sequences of lobe B were then aligned to the HMM and their phylogeny analysed. Parsimony, maximum likelihood and neighbour joining trees were constructed. Fig. 3 shows a consensus maximum likelihood tree generated by Phylip from lobe B sequences that had been bootstrapped 100 times. In each case, the FERM/FA proteins were found to derive from a single node. This supports the hypothesis that FERM/FA region proteins arose from a single ancestor that acquired a FA region adjacent to its FERM domain. Identical conclusions can be drawn from the maximum likelihood and neighbour joining trees (not shown). Note that groups (a) -(e) from Fig. 2 largely cluster together in this tree. As might be predicted, the overall domain structures of the proteins are mirrored in the phylogeny of the FERM domains.

Interestingly, the phosphatase PTN3 [Swiss-Prot:PTN3_HUMAN] does not contain an FA region, yet appears in the FA group. Direct alignment of this sequence with that of the related phosphatase PTN4 [Swiss-Prot:PTN4_HUMAN] leaves a gap where the FA region would be (see Additional file 2); furthermore, probing all possible translations of the genomic sequence of PTN3 with the FA region HMM reveals no FA sequence that might be expressed in splice variants. Maximum parsimony and neighbour joining analyses (not shown) support the positioning of PTN3 in the FA cluster. It seems most likely that PTN3 has lost the FA region present in the common ancestor of PTN3 and PTN4.

### PKC and PKA phosphorylation sites

It has long been established that protein kinases A and C phosphorylate the 16 kDa portion of erythrocyte 4.1R [14,15]. Protein kinase C regulates the membrane binding activity of the 4.1R FERM domain [15,19-21]. Recently Manno et al. [16] have shown that PKC phosphorylates ser-312 in erythrocyte 4.1R (isoform 3 [IPI:IPI00218698]; the equivalent sequence number in [Swiss-Prot:41_human] is ser-521). The site in the 16 kDa portion phosphorylated by PKA is ser-331[14]. Both these residues are part of the FA region.

Ser-312 is not in a known PKC consensus, so it is difficult to predict if equivalent residues in other proteins will be PKC substrates. However, Fig 1 indicates that about half of the FA region proteins have ser at equivalent positions in the alignment.

Ser-331 is in the protein kinase A substrate motif, [KR] [KR]X [ST], and this is conserved in all mammalian "true" 4.1 proteins. It is also conserved in some of the group that lacks known functional domains in their C-termini (e.g. E41L5) and in some of the group of transmembrane proteins (see Fig. 1).

Protein 4.1G [Swiss-Prot:E41L2_human] is also phosphorylated in vivo in the FA region at ser-550 [22]. The kinase that catalyses this phosphorylation has not been identified. Representatives of each of groups (a)-(e) contain ser or thr at this point in the alignment, indicating that this too is a candidate site for phosphorylation in other FA proteins.

## Conclusion

The FA sequence region described here is in all cases immediately adjacent to FERM domains. Based on the chymotrypsin resistance of the 16 kDa 4.1R fragment, and secondary structure predictions, it seems likely that FA regions are folded. The FA region of 4.1R is phosphorylated in vivo by PKA and PKC. The latter phosphorylation is especially important in the red cell, since phosphorylation of ser-312 controls the activity of the adjacent FERM and spectrin-actin binding domains which in turn controls the mechanical properties of the red cell membrane [16]. Since representatives of all the five groups of FA proteins contain ser/thr at sites equivalent to those phosphorylated in the 4.1R and 4.1G FA regions, the possibility arises of a general role of the FA region in regulating the activities of their neighbouring FERM domain.

## Methods

Sequences were retrieved from the UniProt Knowledgebase via the European Bioinformatics Institute (EBI) [23]. BLAST analyses were done with either the BLAST2 server at EBI [24] or the BLAST server at the National Center for Biotechnology Information [25]. The HMMER package [26,27] was used for hidden Markov model analysis; it was run either locally or via a server made available by the Medical Research Council's Rosalind Franklin Centre for Genomics Research [28]. Alignments of sequences to HMMs were done using HMALIGN; alignments were displayed using Chroma [29]. Secondary structure predictions were made using JPRED [30,31]. PSI-Pred [30,32] and Disopred [33,34]. Fold recognition was done using the PSI-PRED and 3D-PSSM [35,36] servers. For phylogenetic analyses, the Phylip package [37] was used.

## Supplementary Material

Additional File 1 **Table 1**. Summary results of searching the Uniprot (Swiss-Prot/Trembl) database with an HMM sequence profile of the FA region. Click here for file

Additional File 2 **Extended alignment of FERM proteins**. The figure shows an alignment of 14 human FERM proteins. The sequences are aligned to 4.1R from Lobe B of the FERM domain, through the 16 kDa fragment. The FA region is shaded blue; Lobe B is shaded red; Lobe C is shaded green. Note that the sequence similarity in the FA region is only found in the FA proteins (41_HUMAN; E4L1_HUMAN; E4L2_HUMAN; E4L3_HUMAN; E4LB_HUMAN; Q9Y4F1; E41LA_HUMAN; Q86WP8). The other proteins (EZRI_HUMAN; TLN1_HUMAN; JAK3_HUMAN; FAK2_HUMAN; PTN3_HUMAN; PTN4_HUMAN) do not contain the FA region, and show no significant similarity in this region. Note too that the sequence similarity in the 16 kDa fragment of 4.1R only extends to the FA regions, and no further. Click here for file
