# Supplementary material for: A FERM-adjacent (FA) region defines a subset of the 4.1 superfamily and is a potential regulator of FERM domain function
Source: BMC Genomics. 2006 Apr 20;7:85. doi: 10.1186/1471-2164-7-85 (PMC1459144; doi:10.1186/1471-2164-7-85)
Supplement: Additional File 1 — Table 1. Summary results of searching the Uniprot (Swiss-Prot/Trembl) database with an HMM sequence profile of the FA region. [file 1471-2164-7-85-S1.pdf]

Additional file.

Table 1. Proteins identified in an HMM search of the Uniprot (Swiss-Prot/Trembl) database

Sequence	Accession	Description	From	To	Score	E-value	N
--------	--------	---	-----	-----	-----	-------	---
41_BOVIN	(Q9N179)	Protein 4.1 (Band 4.1) (P4.1) (4.1R)	286	342	102.8	2.30E-25	1
E41L3_MOUSE	(Q9WV92)	Band 4.1-like protein 3 (4.1B)	402	460	102	4.10E-25	1
Q8BGK4_MOUSE	(Q8BGK4)	Mus musculus 13 days embryo for	402	460	102	4.10E-25	1
Q5DTQ8_MOUSE	(Q5DTQ8)	MKIAA4056 protein (Fragment)	536	592	101.7	5.10E-25	1
41_MOUSE	(P48193)	Protein 4.1 (Band 4.1) (P4.1) (4.1R)	496	552	101.7	5.10E-25	1
Q5R604_PONPY	(Q5R604)	Hypothetical protein DKFZp459B0	285	343	101.4	5.90E-25	1
Q5M7X2_RAT	(Q5M7X2)	Hypothetical protein	396	454	101.4	5.90E-25	1
Q6PA55_XENLA	(Q6PA55)	MGC68473 protein	392	450	101.4	5.90E-25	1
Q9JMB3_RAT	(Q9JMB3)	Type II brain 4.1	397	455	101.4	5.90E-25	1
Q5RBV8_PONPY	(Q5RBV8)	Hypothetical protein DKFZp469E0	198	256	101.4	5.90E-25	1
Q4VAG6_XENTR	(Q4VAG6)	Hypothetical protein (Fragment)	431	489	101.4	5.90E-25	1
Q95JK9_MACFA	(Q95JK9)	Hypothetical protein (Fragment)	398	456	101.4	5.90E-25	1
Q4VB86_HUMAN	(Q4VB86)	Hypothetical protein (Fragment)	406	462	101	7.90E-25	1
41_HUMAN	(P11171)	Protein 4.1 (Band 4.1) (P4.1) (4.1R)	495	551	101	7.90E-25	1
Q5TB35_HUMAN	(Q5TB35)	Erythrocyte membrane protein ba	495	551	101	7.90E-25	1
Q4VB87_HUMAN	(Q4VB87)	Hypothetical protein (Fragment)	300	356	101	7.90E-25	1
Q5TB33_HUMAN	(Q5TB33)	Erythrocyte membrane protein ba	286	342	101	7.90E-25	1
Q59F12_HUMAN	(Q59F12)	Protein 4.1 variant (Fragment)	512	568	101	7.90E-25	1
Q5TB36_HUMAN	(Q5TB36)	Erythrocyte membrane protein ba	460	516	101	7.90E-25	1
41_CANFA	(Q6Q7P4)	Protein 4.1 (Band 4.1) (P4.1) (4.1R)	496	552	100.9	8.30E-25	1
Q7ZXJ6_XENLA	(Q7ZXJ6)	Epb4.1l3 protein (Fragment)	393	451	99.1	3.10E-24	1
Q8IUU7_HUMAN	(Q8IUU7)	EPB41L1 protein (OTTHUMP0000003	381	439	98.8	3.70E-24	1
Q80U34_MOUSE	(Q80U34)	MKIAA0338 protein (Fragment)	409	467	98.8	3.70E-24	1
Q9WTP0_RAT	(Q9WTP0)	Rat brain 4.1(S)	381	439	98.8	3.70E-24	1
Q9WTP1_RAT	(Q9WTP1)	Rat brain 4.1(L)	381	439	98.8	3.70E-24	1
E41L1_MOUSE	(Q9Z2H5)	Band 4.1-like protein 1	381	439	98.8	3.70E-24	1
E41L1_HUMAN	(Q9H4G0)	Band 4.1-like protein 1	381	439	98.8	3.70E-24	1
Q6ZT61_HUMAN	(Q6ZT61)	Hypothetical protein FLJ44927	350	408	98.8	3.70E-24	1
Q96L65_HUMAN	(Q96L65)	Erythrocyte membrane protein 4.	381	439	98.8	3.70E-24	1
Q8K204_MOUSE	(Q8K204)	Epb4.1l1 protein	381	439	98.8	3.70E-24	1
Q4VXN4_HUMAN	(Q4VXN4)	OTTHUMP00000030825	381	439	98.8	3.70E-24	1
Q4VXM8_HUMAN	(Q4VXM8)	OTTHUMP00000030826	284	342	98.8	3.70E-24	1
Q96CV5_HUMAN	(Q96CV5)	Erythrocyte membrane protein	319	377	98.8	3.70E-24	1
41_XENLA	(P11434)	Cytoskeletal protein 4.1	478	534	98.3	5.30E-24	1
Q6DCU8_XENLA	(Q6DCU8)	LOC397741 protein	477	533	98.3	5.30E-24	1
E41L3_HUMAN	(Q9Y2J2)	Band 4.1-like protein 3 (4.1B)	394	452	97.8	7.50E-24	1

Q69ZT8_MOUSE	(Q69ZT8)	MKIAA0987 protein (Fragment)	201	259	97.8	7.50E-24	1
Q9JMB2_RAT	(Q9JMB2)	Type II brain 4.1 minor isoform	397	455	97.8	7.50E-24	1
Q8N4C4_HUMAN	(Q8N4C4)	EPB41L2 protein	503	561	97.5	8.90E-24	1
Q5T4F0_HUMAN	(Q5T4F0)	OTTHUMP00000017197	503	561	97.5	8.90E-24	1
Q59FD8_HUMAN	(Q59FD8)	EPB41L2 protein variant	506	564	97.5	8.90E-24	1
Q68DV2_HUMAN	(Q68DV2)	Hypothetical protein DKFZp781H1	503	561	97.5	8.90E-24	1
E41L2_HUMAN	(O43491)	Band 4.1-like protein 2	503	561	97.5	8.90E-24	1
Q5RC68_PONPY	(Q5RC68)	Hypothetical protein DKFZp459F2	495	553	97.5	9.00E-24	1
E41L2_MOUSE	(O70318)	Band 4.1-like protein 2	496	556	96.9	1.40E-23	1
Q8C928_MOUSE	(Q8C928)	Mus musculus 7 days neonate cer	488	548	96.9	1.40E-23	1
Q80UE5_MOUSE	(Q80UE5)	Protein 4.1G	496	556	96.9	1.40E-23	1
Q80UE3_MOUSE	(Q80UE3)	Protein 4.1G	496	556	96.9	1.40E-23	1
Q811B2_MOUSE	(Q811B2)	Protein 4.1G	496	556	96.9	1.40E-23	1
Q811C0_MOUSE	(Q811C0)	4.1G protein (Fragment)	234	294	96.9	1.40E-23	1
Q80UE4_MOUSE	(Q80UE4)	Protein 4.1G	496	556	96.9	1.40E-23	1
Q5U540_XENLA	(Q5U540)	Epb4.1l3 protein (Fragment)	285	348	96.3	2.10E-23	1
Q5R6C1_PONPY	(Q5R6C1)	Hypothetical protein DKFZp459E1	394	452	95.9	2.70E-23	1
Q8C8P2_MOUSE	(Q8C8P2)	Mus musculus adult retina cDNA,	381	438	92.8	2.30E-22	1
Q68EI5_BRARE	(Q68EI5)	Zgc:92004	374	432	90.5	1.20E-21	1
E41LB_MOUSE	(Q9JMC8)	Band 4.1-like protein 4B	375	435	88	6.60E-21	1
Q5VX11_HUMAN	(Q5VX11)	Erythrocyte membrane protein ba	375	435	85.2	4.70E-20	1
E41LB_HUMAN	(Q9H329)	Band 4.1-like protein 4B	361	421	85.2	4.70E-20	1
Q59GC2_HUMAN	(Q59GC2)	Erythrocyte membrane protein ba	78	138	85.2	4.70E-20	1
Q5VX12_HUMAN	(Q5VX12)	Erythrocyte membrane protein ba	375	435	85.2	4.70E-20	1
Q9NX84_HUMAN	(Q9NX84)	Hypothetical protein FLJ20381	297	357	85.2	4.70E-20	1
Q91VS8_MOUSE	(Q91VS8)	FERM, RhoGEF and pleckstrin domains	329	386	83.2	1.90E-19	1
Q8K2R0_MOUSE	(Q8K2R0)	Farp1 protein (Fragment)	112	168	83	2.20E-19	1
Q8IZ12_HUMAN	(Q8IZ12)	EPB41L5 protein	333	388	80.7	1.10E-18	1
Q4ZG32_HUMAN	(Q4ZG32)	Hypothetical protein EPB41L5	333	388	80.7	1.10E-18	1
E41L5_HUMAN	(Q9HCM4)	Band 4.1-like protein 5	333	388	80.7	1.10E-18	1
Q7Z5S1_HUMAN	(Q7Z5S1)	Erythrocyte membrane protein ba	333	388	80.7	1.10E-18	1
Q6IQ29_HUMAN	(Q6IQ29)	FARP1 protein	325	381	80.5	1.20E-18	1
Q9Y4F1_HUMAN	(Q9Y4F1)	CDEP (FERM, RhoGEF, and pleckst	325	381	80.5	1.20E-18	1
Q5RAB8_PONPY	(Q5RAB8)	Hypothetical protein DKFZp469C0	325	381	80.5	1.20E-18	1
Q9MYU8_CANFA	(Q9MYU8)	Band4.1-like5 protein	333	388	80.3	1.30E-18	1
Q58CU2_BOVIN	(Q58CU2)	Erythrocyte membrane protein ba	333	388	79.6	2.30E-18	1
Q69ZG8_MOUSE	(Q69ZG8)	MKIAA1548 protein (Fragment)	383	438	79.3	2.80E-18	1
Q99KZ8_MOUSE	(Q99KZ8)	Epb4.1l5 protein (Mus musculus)	333	388	79.3	2.80E-18	1
Q8BSC9_MOUSE	(Q8BSC9)	Mus musculus 12 days embryo emb	333	388	79.3	2.80E-18	1
Q8BGS1_MOUSE	(Q8BGS1)	Mus musculus 9.5 days embryo pa	333	388	79.3	2.80E-18	1
Q5FVG2_RAT	(Q5FVG2)	Erythrocyte protein band 4.1-like 5	333	388	79.2	3.00E-18	1
Q6NYK4_BRARE	(Q6NYK4)	Erythrocyte membrane protein band 4.1-like 5	340	392	77.1	1.30E-17	1
Q6PNC5_BRARE	(Q6PNC5)	Mosaic eyes	333	385	77.1	1.30E-17	1
Q86LF2_DROME	(Q86LF2)	LD13416p	206	264	76.7	1.60E-17	1
Q9W0R3_DROME	(Q9W0R3)	CG1228-PA, isoform A	302	360	76.7	1.60E-17	1
Q8IRJ0_DROME	(Q8IRJ0)	CG1228-PB, isoform B	141	199	76.7	1.60E-17	1
Q8WSF2_DROME	(Q8WSF2)	Split central complex	324	382	76.7	1.60E-17	1

Q8JG61_BRARE	(Q8JG61)	Protein 4.1	286	340	76.3	2.20E-17	1
Q8BQQ4_MOUSE	(Q8BQQ4)	Protein tyrosine phosphatase, non-receptor type 4	319	376	76.1	2.60E-17	1
PTN4_HUMAN	(P29074)	Tyrosine-protein phosphatase, non-receptor type 4	319	376	76.1	2.60E-17	1
Q8AW25_BRARE	(Q8AW25)	Novel protein similar to KIAA15	262	314	75.6	3.60E-17	1
Q61JK1_CAEBR	(Q61JK1)	Hypothetical protein CBG09764	319	376	75.5	3.80E-17	1
PTP1_CAEEL	(P28191)	Tyrosine-protein phosphatase 1	319	376	75.5	3.80E-17	1
Q8N3Y5_HUMAN	(Q8N3Y5)	FRMD3 protein (OTTHUMP00000021541)	125	185	75.5	3.80E-17	1
Q5R803_PONPY	(Q5R803)	Hypothetical protein DKFZp469K0	319	379	75.5	3.80E-17	1
Q53EP2_HUMAN	(Q53EP2)	FERM domain containing 3 variant	303	363	75.5	3.80E-17	1
Q86WP8_HUMAN	(Q86WP8)	Protein band 4.1-like protein 4	275	335	75.5	3.80E-17	1
Q8IW02_HUMAN	(Q8IW02)	FERM domain containing 3	303	363	75.5	3.80E-17	1
Q8N9L2_HUMAN	(Q8N9L2)	Hypothetical protein FLJ36962	223	283	75.5	3.80E-17	1
Q9WU22_MOUSE	(Q9WU22)	Testis-enriched protein tyrosine phosphatase	319	376	74.5	7.40E-17	1
Q9VFU8_DROME	(Q9VFU8)	CG9764-PA (LD33734p)	348	401	73.6	1.50E-16	1
Q95RU5_DROME	(Q95RU5)	LD11531p	105	158	73.6	1.50E-16	1
Q86PA5_DROME	(Q86PA5)	LD27491p	8	67	73.3	1.80E-16	1
Q5SPU1_MOUSE	(Q5SPU1)	FERM domain containing 3	329	389	72	4.40E-16	1
Q8BHD4_MOUSE	(Q8BHD4)	Similar to 2310003M01RIK PROTEIN	319	379	72	4.40E-16	1
Q5SPU0_MOUSE	(Q5SPU0)	FERM domain containing 3	329	389	72	4.40E-16	1
Q7QB86_ANOGA	(Q7QB86)	ENSANGP00000011297 (Fragment)	273	326	70.9	9.40E-16	1
Q8WU27_HUMAN	(Q8WU27)	FARP2 protein	329	386	70.5	1.20E-15	1
O94887_HUMAN	(O94887)	KIAA0793 protein (Fragment)	330	387	70.5	1.20E-15	1
Q6NSM6_BRARE	(Q6NSM6)	Zgc:85963	364	425	69.5	2.40E-15	1
Q6NRP9_XENLA	(Q6NRP9)	MGC83117 protein	319	379	69.4	2.60E-15	1
Q6DCH9_XENLA	(Q6DCH9)	MGC83117 protein	319	379	69.4	2.60E-15	1
E41L4_BRARE	(O57457)	Band 4.1-like protein 4 (Nbl4 protein)	307	362	69.2	2.90E-15	1
Q6P982_BRARE	(Q6P982)	Epb4.1l4 protein	307	362	69.2	2.90E-15	1
Q9V9Y3_DROME	(Q9V9Y3)	CG11339-PA	284	337	67.6	9.10E-15	1
Q8JFS7_BRARE	(Q8JFS7)	Novel protein tyrosine phosphat	319	376	67.1	1.30E-14	1
Q9GP93_CAEEL	(Q9GP93)	Ferm domain family protein 2	329	390	66.9	1.50E-14	1
Q6DJ46_XENTR	(Q6DJ46)	Erythrocyte membrane protein band 4.1 like 4A	307	364	66.7	1.70E-14	1
Q6NRR3_XENLA	(Q6NRR3)	MGC82292 protein	307	369	65.8	3.10E-14	1
41_DROME	(Q9V8R9)	Protein 4.1 homolog (Coracle protein)	319	375	64.4	8.40E-14	1
Q8MLI7_DROME	(Q8MLI7)	CG11949-PD, isoform D	319	375	64.4	8.40E-14	1
Q7T0Z9_XENLA	(Q7T0Z9)	Epb4.1l5-prov protein	333	384	64.3	9.10E-14	1
Q61L91_CAEBR	(Q61L91)	Hypothetical protein CBG09029	346	407	63	2.20E-13	1
Q91VX6_MOUSE	(Q91VX6)	Epb4.1l4a protein	307	364	61.9	4.60E-13	1
E41LA_HUMAN	(Q9HCS5)	Band 4.1-like protein 4A (NBL4 protein)	307	364	60.4	1.30E-12	1
O17905_CAEEL	(O17905)	Hypothetical protein frm-3	334	391	57.6	9.10E-12	1
Q610U6_CAEBR	(Q610U6)	Hypothetical protein CBG17296	334	391	57.6	9.10E-12	1
Q9XV97_CAEEL	(Q9XV97)	Hypothetical protein frm-1	317	368	49.4	2.80E-09	1
Q7YSV5_CAEEL	(Q7YSV5)	Hypothetical protein frm-1	328	379	49.4	2.80E-09	1
Q9TVI9_CAEEL	(Q9TVI9)	Hypothetical protein frm-1	317	368	49.4	2.80E-09	1
O18673_CAEEL	(O18673)	Hypothetical protein frm-1	317	368	49.4	2.80E-09	1
Q86P10_DROME	(Q86P10)	RE05264p	224	280	49.2	3.10E-09	1
Q9VKY7_DROME	(Q9VKY7)	CG5022-PA	304	360	49.2	3.10E-09	1
Q7PTB6_ANOGA	(Q7PTB6)	ENSANGP00000007560 (Fragment)	330	385	48.5	5.10E-09	1

E41LA_MOUSE	(P52963)	Band 4.1-like protein 4A (NBL4 protein)	307	362	47.6	9.50E-09	1
Q7PVA6_ANOGA	(Q7PVA6)	ENSANGP00000009134	316	370	42.5	3.20E-07	1
Q9VN68_DROME	(Q9VN68)	CG31536-PC, isoform C (SD09116p	360	404	41.8	5.40E-07	1
Q61LU2_CAEBR	(Q61LU2)	Hypothetical protein CBG08758	323	377	40.2	1.60E-06	1
Q5JX99_HUMAN	(Q5JX99)	Novel protein	167	230	39.3	2.90E-06	1
Q6ZUT3_HUMAN	(Q6ZUT3)	Hypothetical protein FLJ43346	287	350	39.3	2.90E-06	1
Q5JU84_HUMAN	(Q5JU84)	OTTHUMP00000021543 (Fragment)	88	137	37.4	1.10E-05	1
Q7Q844_ANOGA	(Q7Q844)	ENSANGP00000018824 (Fragment)	304	361	35.9	3.20E-05	1
Q8NBG4_HUMAN	(Q8NBG4)	Hypothetical protein FLJ33253	290	343	35.4	4.50E-05	1
Q8BXT6_MOUSE	(Q8BXT6)	Mus musculus adult retina cDNA,	216	269	35.4	4.50E-05	1
Q8C0K0_MOUSE	(Q8C0K0)	Mus musculus adult male thymus cDNA	216	269	35.4	4.50E-05	1
Q6PFH6_MOUSE	(Q6PFH6)	A930004K21Rik protein	216	269	35.4	4.50E-05	1
Q6P5H6_MOUSE	(Q6P5H6)	RIKEN cDNA A930004K21	305	358	35.4	4.50E-05	1
Q7Z6J6_HUMAN	(Q7Z6J6)	MGC14161 protein	305	358	35.4	4.50E-05	1
Q52L19_XENLA	(Q52L19)	Hypothetical protein (Fragment)	116	179	32.2	0.00042	1
Q8GUI3_ARATH	(Q8GUI3)	Hypothetical protein (At5g61270)	354	412	32	0.00048	1
Q7PX95_ANOGA	(Q7PX95)	ENSANGP00000020346	341	379	22.4	0.069	1
Q580X3_HUMAN	(Q580X3)	Hypothetical protein PTPN4 (Fragment)	273	310	22	0.077	1
Q7PXW8_ANOGA	(Q7PXW8)	ENSANGP00000017385 (Fragment)	267	319	15.6	0.48	1

The table shows the results of an HMM search for sequences that contain the FA domain. The database used was the Uniprot Knowledgebase (2,024,194 entries, indexed 03-Jul-2005).
