# Supplementary material for: A FERM-adjacent (FA) region defines a subset of the 4.1 superfamily and is a potential regulator of FERM domain function
Source: BMC Genomics. 2006 Apr 20;7:85. doi: 10.1186/1471-2164-7-85 (PMC1459144; doi:10.1186/1471-2164-7-85)
Supplement: Additional File 2 — Extended alignment of FERM proteins. The figure shows an alignment of 14 human FERM proteins. The sequences are aligned to 4.1R from Lobe B of the FERM domain, through the 16 kDa fragment. The FA region is shaded blue; Lobe B is shaded red; Lobe C is shaded green. Note that the sequence similarity in the FA region is only found in the FA proteins (41_HUMAN; E4L1_HUMAN; E4L2_HUMAN; E4L3_HUMAN; E4LB_HUMAN; Q9Y4F1; E41LA_HUMAN; Q86WP8). The other proteins (EZRI_HUMAN; TLN1_HUMAN; JAK3_HUMAN; FAK2_HUMAN; PTN3_HUMAN; PTN4_HUMAN) do not contain the FA region, and show no significant similarity in this region. Note too that the sequence similarity in the 16 kDa fragment of 4.1R only extends to the FA regions, and no further. [file 1471-2164-7-85-S2.pdf]

Additional file 2. Alignment of a selection of human FERM proteins from Lobe 2 through the 16 kDa fragment of 4.1R

FERM Lobe B

```
PTN3_HUMAN      QTRHLYFLQLKMDICEGRLTCPLNSAVVLASYAVQSHFGDYNSSIHHPGYLSDSHFIPDQ--------NEDFLTKVESLHEQHS-GLKQSEAESCYINIARTLDFYGVELHSGRDLHNLD
PTN4_HUMAN      YTRYQYFLQIKQDILTGRLPCPSNTAALLASFAVQSELGDYDQSENLSGYLSDYSFIPNQ--------PQDFEKEIAKLHQQHI-GLSPAEAEFNYLNTARTLELYGVEFHYARDQSNNE
41_HUMAN        ITRYYLCLQLRQDIVAGRLPCSFATLALLGSYTIQSELGDYDPELHGVDYVSDFKLAPNQ--------TKELEEKVMELHKSYR-SMTPAQADLEFLENAKKLSMYGVDLHKAKDLEGVD
E4L1_HUMAN      ITRYYLCLQLRADIITGRLPCSFVTHALLGSYAVQAELGDYDAEEHVGNYVSELRFAPNQ--------TRELEERIMELHKTYRGMTPGEAEIHFLENAKKLSMYGVDLHH-AKDSEGID
E4L2_HUMAN      ITRYFLCLQLRQDIASGRLPCSFVTHALLGSYTLQAELGDYDPEEHGSIDLSEFQFAPTQ--------TKELEEKVAELHKTHRGLSPAQADSQFLENAKRLSMYGVDLHH-AKDSEGVD
E4L3_HUMAN      ITRYYLCLQLRDDIVSGRLPCSFVTLALLGSYTVQSELGDYDPDECGSDYISEFRFAPN---------TKELEDKVIELHKSHRGMTPAEAEMHFLENAKKLSMYGVDLHH-AKDSEGVE
E4LB_HUMAN      FTRYLFVLQLRHDILSGKLKCPYETAVELAALCLQAELGECELPEHTPELVSEFRFIPNQ--------TEAMEFDIFQRWKECRGKSPAQAELSYLNKAKWLEMYGVDMHVVRGRDGCEY
Q9Y4F1          LTRYLFALQVKQDLAQGRLTCNDTSAALLISHIVQSEIGDFDEALDR-EHLAKNKYIPQQ---------DALEDKIVEFHHNHI-GQTPAESDFQLLEIARRLEMYGIRLHPAKDREGTK
E41LA_HUMA      ITRYQFFLQVKQDVLQGRLPCPVNTAAQLGAYAIQSELGDYDPYKHTAGYVSEYRFVPDQ--------KEELEEAIERIHKTLM-GQIPSEAELNYLRTAKSLEMYGVDLHPVYGENKSE
Q86WP8          LTRYLLYLQIKRDIFHGRLLCSFSDAAYLGACIVQAELGDYDPDEHPENYISEFEIFPKQ--------SQKLERKIVEIHKNELRGQSPPVAEFNLLLKAHTLETYGVDPHPCKDSTGTT
EZRI_HUMAN      ITQKLFFLQVKEGILSDEIYCPPETAVLLGSYAVQAKFGDYNKEVHKSGYLSSERLIPQRVMDQHKLTRDQWEDRIQVWHAEH-RGMLKDNAMLEYLKIAQDLEMYGINYFEIKNKKGTD
TLN1_HUMAN      VQLNLLYVQARDDILNGSHPVSFDKACEFAGFQCQIQFGPHNEQKHKAGFLDLKDFLPKEYVKQKGERKIFQAHKNCGQMSEIEAKVRYVKLARSLKTYGVSFFLVKEKMKGKNKLVPRL
JAK3_HUMAN      LEHLFAQHRSDLVSGRLPVGLSLKEQGECLSLAVLDLARMAREQAQRPGELLKTVSYKACLPPSLRDLIQGLSFVTRRRIRRTVRRALRRVAACQADRHSLMAKYIMDLERLDPAGAAET
FAK2_HUMAN      TTLLYFYQQLRNDYMQRYASKVSEGMALQLGCLELRRFFKDMPHNALDKKSNFELLEKEVGLDLFFPKQMQENLKPKQFRKMIQQTFQQYASLREEECVMKFFNTLAGFANIDQETYRCE
Consensus/51%   lTRYbbbLQl+pDIlsGRLsCsbsTAALLtSasVQtELGDYDspcHhssYlS-b+FbPsQ........sccLEcKlbclHKpap.sbsssstbppbbpst+pbpbbtspbH.s+Dppts-
```

FERM Lobe C

FERM domain

16 kDa fragment

```
PTN3_HUMAN      LMIGIASAGVAVYRKYICTSF---YPWVNILKISFKRKKFFIHQRQKQAESREHIVAFNMLNY--RSCKNLWKSCVEHHTFFQAKKLLPQEKNVL-SQYWTMGSR---------------
PTN4_HUMAN      IMIGVMSGGILIYKNRVRMNT---FPWLKIVKISFKCKQFFIQLRKELHESRETLLGFNMVNY--RACKNLWKACVEHHTFFRLDRPLPPQKNFF-AHYFTLGSKFRYCGRTEVQSVQYG
41_HUMAN        IILGVCSSGLLVYKDKLRINR---FPWPKVLKISYKRSSFFIKIRPGEQEQYESTIGFKLPSY--RAAKKLWKVCVEHHTFFRL-TSTDTIPK---SKFLALGSKFRYSGRTQAQTRQAS
E4L1_HUMAN      IMLGVCANGLLIYRDRLRINR---FAWPKILKISYKRSNFYIKIRPGEYEQFESTIGFKLPNH--RSAKRLWKVCIEHHTFFRLVSP--EPPPK---GFLVMGSKFRYSGRTQAQTRQAS
E4L2_HUMAN      IKLGVCANGLLIYKDRLRINR---FAWPKILKISYKRSNFYIKVRPAELEQFESTIGFKLPNH--RAAKRLWKVCVEHHTFYRLVSP--EQPPK--AKFLTLGSKFRYSGRTQAQTRQAS
E4L3_HUMAN      IMLGVCASGLLIYRDRLRINR---FAWPKVLKISYKRNNFYIKIRPGEFEQFESTIGFKLPNH--RAAKRLWKVCVEHHTFFRLLLP--EAPPK---KFLTLGSKFRYSGRTQAQTRRAS
E4LB_HUMAN      SLGLTPTGILIFEGANKIGLF---FWPKITKMDFKKSKLTLVVVEDDDQGR-EQEHTFVFRLDSARTCKHLWKCAVEHHAFFRLRTPGNSKSNR--SDFIRLGSRFRFSGRTEYQATHGS
Q9Y4F1          INLAVANTGILVFQGFTKINA---FNWAKVRKLSFKRKRFLIKLRPDANSAYQDTLEFLMASR--DFCKSFWKICVEHHAFFRL-FEEPKPKPK--PVLFSRGSSFRFSGRTQKQVLDYV
E41LA_HUMA      YFLGLTPVGVVVYKNKKQVGK---YFWPRITKVHFKETQF--ELRVLGKDCNETSFFFEARSK--TACKHLWKCSVEHHTFFRMPENESNSLSRKLSKFGSIRYKHRYSGRTALQMSRDL
Q86WP8          TFLGFTAAGFVVFRGNKRIHL---IKWPDVCKLKFEGKTFYV-----IGTQKEKKAMLAFHTSTPAACKHLWKCGVENQAFYKYA-KSSQIKTVSSSKIFFKGSRFRYSGKVAKEVVEAS
EZRI_HUMAN      LWLGVDALGLNIYEKDDKLTPKIGFPWSEIRNISFNDKKFVIK----PIDKKAPDFVFYAPRLRINKRILQLCMGNHELY-MRRRKPDTIEVQQMKAQAREEKHQKQLERQQLETEKKRR
TLN1_HUMAN      LGITKECVMRVDEKTKEVIQEWNLTNIKRWAASPKSFTLDFGDYQDGYYSVQTTEGEQIAQLIAGYIDIILKKKKSKDHFGLEGDEESTMLEDSVSPKKSTVLQQQYNRVGKVEHGSVAL
JAK3_HUMAN      FHVGLPGALGGHDGLGLLRVAGDGGIAWTQGEQEVLQPFCDFPEIVDISIKQAPRVGPAGEHRLVTVTRTDNQILEAEFPGLPEALSFVALVDGYFRLTTDSQHFFCKEVAPPRLLEEVA
FAK2_HUMAN      LIQGWNITVDLVIGPKGIRQLTSQDAKPTCLAEFKQIRSIRCLPLEEGQAVLQLGIEGAPQALSIKTSSLAEAENMADLIDGYCRLQGEHQGSLIIHPRKDGEKRNSLPQIPMLNLEARR
Consensus/51%   lbLGlstsGlllY+s+b+lsb...FsWs+lbKlSaKcppFaIclRssbb-pbEsslsFbb.sp..+tsKpLWKssVEHHsFFRb.ps.spbssp..tcabsbGS+FRaSGRTbbQsppst
```

FA

```
PTN3_HUMAN      ------NTKKSVNNQYCKKVIGGMVWNPAMRRSLSVEHLETKSLPSRS-PPITPNWRSPRLRHEIRKPRHSSADNLANEMTYITETEDVFYTYKGSL
PTN4_HUMAN      KEKAN-KDRVFARSPSKPLARKLMDWEVVSRNSISDDRLETQSLPSRS-PPGTPNHRNSTFTQEGTRLRPSSVGHLVDHMVH-TSPSEVFVNQRSPS
41_HUMAN        ALID-RPAPHFERTASKRASRSLDGAAAVDSADRSPRPTSAPAITQG-QVAE--GGVLDASAKKTVVPKAQKETVKAEVKKEDEPPEQAEPEPTEAW
E4L1_HUMAN      ALID-RPAPFFERSSSKRYTMSRSLDG----AEFSRPASVSENHDAGPDGDKRDEDGESGGQRSEAEEGEVRTPTKIKELKPEQETTPRHKQEFLDK
E4L2_HUMAN      TLID-RPAPHFERTSSKRVSRSLDG-APIGVMDQSLMKDFPGAAGEISAYGPGLVSIAVVQDGDGRREVRSPTKAPHLQLIEGKKNSLRVEGDNIYV
E4L3_HUMAN      ALID-RPAPYFERSSSKRYTMSRSLDGEVGTGQYATTKGISQTNLITTVTPEKKAEEERDEEEDKRRKGEEVTP--ISAIRHEGKSPGLGTDSCPLS
E4LB_HUMAN      RL---RRTSTFERKPSKRYPSRRHSTFKASNPVIAAQLCSKTNPEVHNYQPQYHPNIHPSQPRWHPHSPNVSYPLPSPVLSSSDRLPFGIEENGGTP
Q9Y4F1          KE-GGHKKVQFERKHSKIHSIRSLASQPTELNSEVLEQSQQSTSLTFG-EGAESPGGQSCRRGKEPKVSAGEPGSHPSPAPRRSPAGNKQADGAASA
E41LA_HUMA      SIQLPRPDQNVTRSRSKTYPKRIAQTQPAESNTISRITANMENGENEG-TIKIIAPSPVKSFKKAKNENSPDTQRSKSLMHSWEENGPQSGLYNSPS
Q86WP8          SKIQREPPE-VHRANITQSRSS-HSLNKQLIINMEPLQPLLPSPSEQEEELPLGEGVPLPKEENISAPLISSSPVKAAREYEDPPSEEEDKIKEEPL
EZRI_HUMAN      ETVEREKEQMMREKEELMLRLQDYEEKTKKAERELSEQIQRALQLEEERKRAQEEAERLEADRMAALRAKEELERQAVDQIKSQEQLAAELAEYTAK
TLN1_HUMAN      PAIMRSGASGPENFQVGSMPPAQQQITSGQMHRGHMPPLTSAQQALTGTINSSMQAVQAAQATLDDFDTLPPLGQDAASKAWRKNKMDESKHEIHSQ
JAK3_HUMAN      EQCHGPITLDFAINKLKTGGSRPGSYVLRRSPQDFDSFLLTVCVQNPLGPDYKGCLIRRSPTGTFLLVGLSRPHSSLRELLATCWDGGLHVDGVAVT
FAK2_HUMAN      SHLSESCSIESDIYAEIPDETLRRPGGPQYGIAREDVVLNRILGEGFFGEVYEGVYTNHKGEKINVAVKTCKKDCTLDNKEKFMSEAVIMKNLDHPH
Consensus/51%   sblp.css.pF-RssSKpbsppbpshsssspsssbtsppspssss.sps..sbp.sshpssppcphspsspssssspsspbbcpppsssbpbspsssp
```
